# A microemulsion high-performance liquid chromatography (MELC) method for the separation and determination of hydrolyzed tenuifolin in Radix Polygalae

**DOI:** 10.1038/s41598-019-55416-z

**Published:** 2019-12-13

**Authors:** Hui Yan, Zhuan-Di Zheng, Hong-Fei Wu, Xiao-Chuang Liu, An Zhou

**Affiliations:** 1 Department of Pharmacy, Anhui University of Chinese Medicine, Hefei, Anhui 230012 P. R. China; 2 Anhui Province Key Laboratory of Chinese Medicinal Formula, Hefei, Anhui 230012 P. R. China; 3 The First Affiliated Hospital of Anhui University of Chinese Medicine, Hefei, 230026 P. R. China

**Keywords:** Liquid chromatography, Analytical chemistry, Green chemistry

## Abstract

Tenuifolin was used as a reliable chemical marker for the quality control of Radix Polygalae. The determination of tenuifolin is challenging because the analyte molecule lacks a suitable chromophore. The aim of this study was to establish a microemulsion high-performance liquid chromatography (MELC) method which is robust and sensitive, and can separate and determine tenuifolin in Radix Polygalae using an oil-in-water (O/W) microemulsion mobile phase. The separations were performed on a C18 (4.6 × 250 mm, 5 μm) column at 25 °C using a flow rate of 1.0 mL/min, and an ultraviolet detection wavelength of 210 nm. The microemulsion mobile phase comprised 2.8% (w/v) sodium dodecyl sulfate (SDS), 7.0% (v/v) n-butanol, 0.8% (v/v) n-octane and 0.1% (v/v) aqueous orthophosphate buffer (H3PO4). The linearity analysis of tenuifolin showed a correlation coefficient of 0.9923 in the concentration range of 48.00–960.00 µg/mL. The accuracy of the method based on three concentration levels ranged from 96.23% to 99.28%; the limit of detection (LOD) was 2.34 µg/mL, and the limit of quantification (LOQ) was 6.76 µg/mL. The results of our study indicated that the optimized MELC method was sensitive and robust, and can be widely applied for the separation and determination of tenuifolin in Radix Polygalae.

## Introduction

Radix Polygalae, named “YuanZhi” in traditional Chinese medicine, is the root of *Polygala tenuifolia* Willd. or *Polygala sibirica* L^1^. Modern pharmacological studies indicate that Radix Polygalae possesses various biological activities against certain ailments, such as cerebrovascular diseases, senile dementia and depression^2–4^. Moreover, some researchers found that Radix Polygalae could effectively suppress the consolidation and reinstatement of fear memories^5^. With the widespread clinical application of Radix Polygalae^6,7^, it is important to control the quality of Radix Polygalae. Tenuifolin is considered one of the main effective components of Radix Polygalae, which is beneficial for treating certain diseases of the central nervous system^8,9^. In addition, tenuifolin belongs to the chemical class of secondary saponins and exhibits stable chemical properties^9^. Therefore, tenuifolin was applied for the evaluation of the quality of Radix Polygalae in this study. To better control the quality of Radix Polygalae, there was a need to establish a method to separate and determine the concentration of tenuifolin.

**Figure 1:**
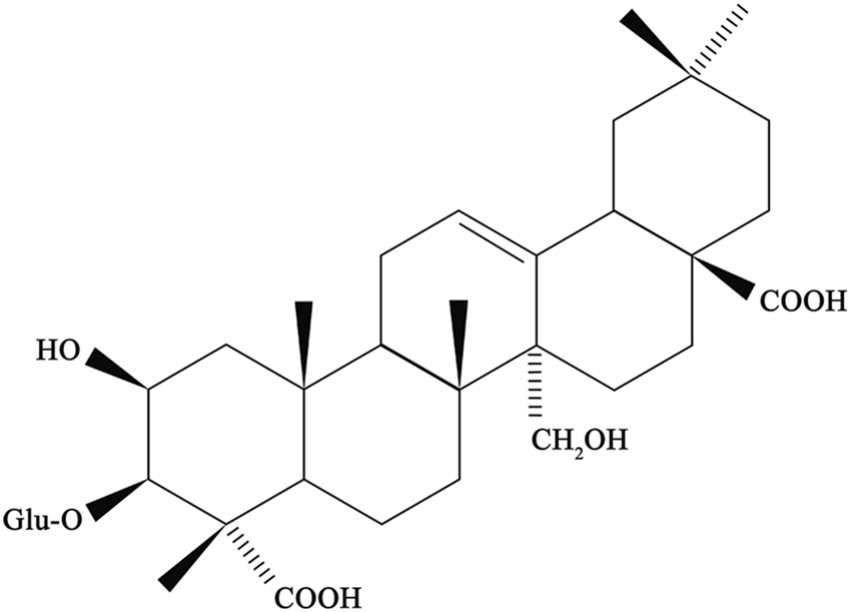
The chemical structure of tenuifolin.

Over the years, various quantitative analysis methods for determination of tenuifolin including high performance liquid chromatography (HPLC)^10^, high performance capillary electrophoresis (HPCE)^11^ and liquid chromatography with tandem mass spectrometry (LC-MS/MS)^12^ have been developed. Due to poor ultraviolet absorptivity, tenuifolin can only be analyzed at low-ultraviolet wavelength detection with HPLC (210 nm, chemical structure of tenuifolin is shown in Fig. 1). CAD usually requires higher maintenance and less precision. LC-MS/MS has high sensitivity and specificity, however, It is generally used for *in vivo* drug analysis on the basis of economical and applicable principles. Therefore, it is necessary to develop a new effective, sensitive, selective, and accurate analytical method for the determination of tenuifolin in Radix Polygalae.

The microemulsion high-performance liquid chromatography (MELC) method is a relatively new chromatographic technique^13^, that utilizes microemulsion as the mobile phase and has been shown to be suitable for the separation of pharmaceutical compounds using both isocratic and gradient elution modes. The MELC method has many advantages that are superior to conventional chromatography, such as less organic solvent and lower cost^14^. MELC has comparable or higher selectivity and separation efficiency than conventional HPLC systems^15^. The microemulsion formulation has a crucial effect on the separation and retention. Additionally, MELC is usful for low-ultraviolet detection wavelengths (190 nm) with high sensitivity for weak chromophore compounds. When the chromophore was limited, the detection was superior to conventional HPLC^16^. MELC has been successfully applied to determine drugs in pharmaceutical dosage form^17,18^, to analyze biological samples^19,20^, and to separate and determine content in Chinese traditional medicine^21,22^.

In the present work, we investigated the possibility of separation and determination of tenuifolin in Radix Polygalae by reversed-phase high-performance liquid chromatography using an oil-in-water microemulsion as the mobile phase. Moreover, some critical parameters affecting the separation and determination selectivity of the MELC system, such as the surfactant, the cosurfactant, the oil phase and the temperature, were studied in detail.

## Results and Discussion

### Particle size of the mobile phase

**Figure 2:**
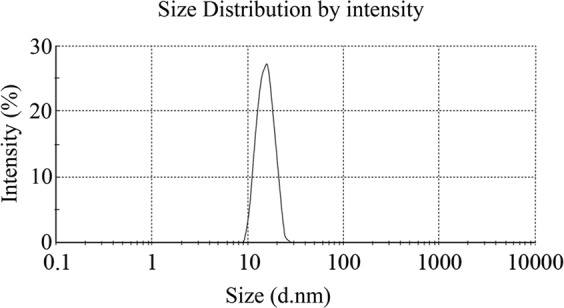
Size distribution (by intensity) of microemulsion mobile phase. The mobile phase consists of 2.8% (w/v) SDS, 7.0% (v/v) n-butanol, 0.8% (v/v) n-octane and 0.1% (v/v) H_3_PO_4_ measured by the particle size analyzer.

The average particle size of the microemulsion was detected by three repeated assays. The particle size of the microemulsion mobile phase was 15.57 ± 2.95 nm (Fig. 2). In addition, the microemulsion system remained relatively stable during the experiment.

### Optimization of MELC conditions

#### Selection of surfactant type and concentration

**Figure 3:**
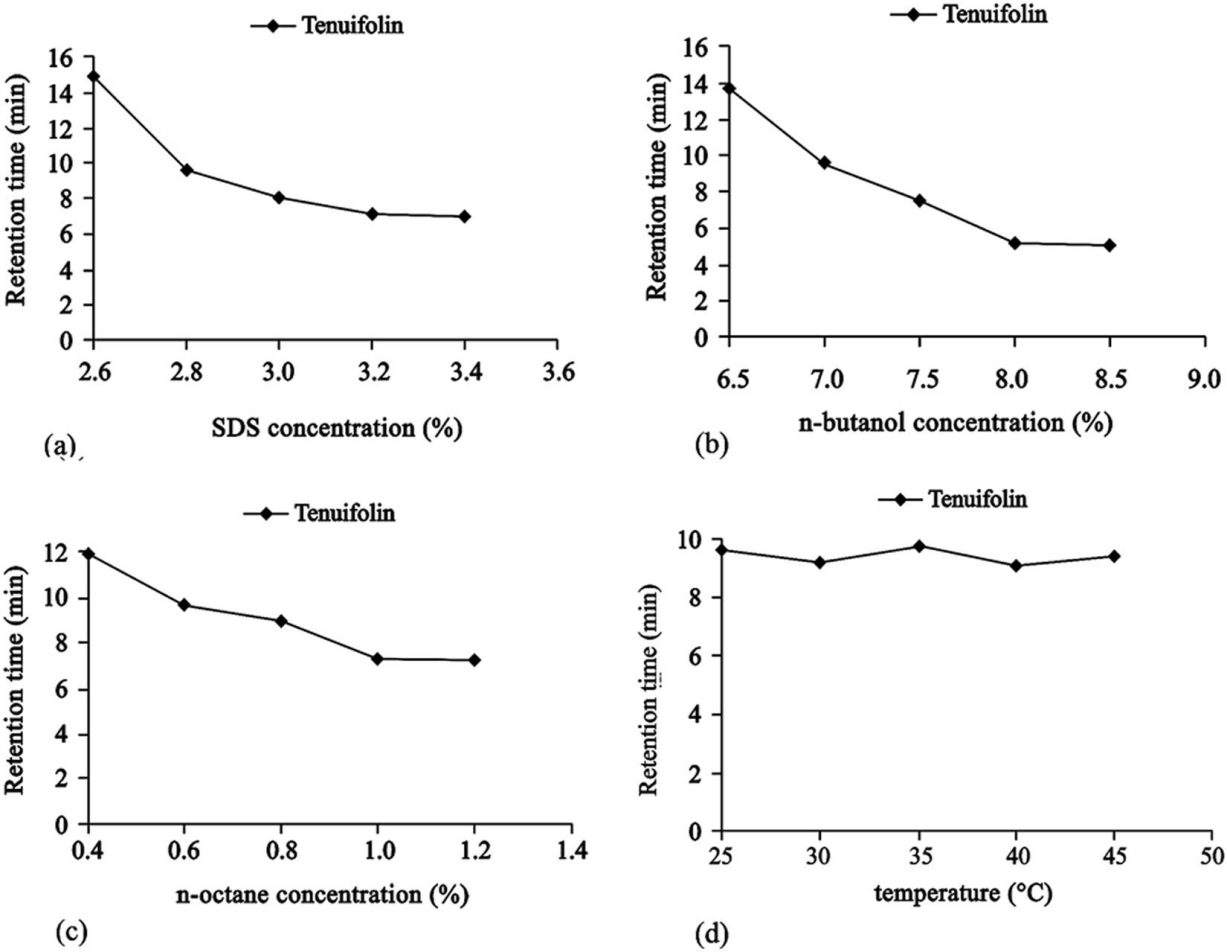
(**a**) Effect of SDS concentration (%, w/v) in the microemulsion on the retention time of tenuifolin using microemulsion mobile phases consisting of different concentrations of SDS, 7.0% v/v n-butanol and 0.8% v/v n-octane. (**b**) Effect of n-butanol concentration (%, v/v) in the microemulsion on the retention time of tenuifolin using microemulsion mobile phases consisting of different concentrations of n-butanol, 2.8% w/v SDS and 0.8% v/v n-octane. (**c**) Effect of n-octane concentration (%, v/v) in the microemulsion on the retention time of tenuifolin using microemulsion mobile phases consisting of different concentrations of n-octane, 2.8% v/v SDS and 7.0% v/v n-butanol. (**d**) Effect of column temperature (°C) on the retention time of tenuifolin using microemulsion mobile phases consisting of 2.8% v/v SDS, 7.0% v/v n-butanol and 0.8% v/v n-octane.

The surfactant in the mobile phase has a remarkable effect on the separation selectivity in the mobile phase. The surfactant can change the surface of the stationary phase because it can adhere to the porous stationary phase, which has a direct influence on the retention of solutes^23^. In this study, SDS, CTAB and Tween-80 were tested for using in the oil-in-water microemulsion. Both CTAB and Tween-80 failed to offer a better baseline resolution and separation for tenuifolin. Therefore, SDS was the optimal surfactant in this study. Five concentrations of SDS were studied (Fig. 3a). It was found that the retention time of tenuifolin decreased upon increasing the concentration of SDS from 2.6 to 3.2% w/v. This showed that SDS may have changed the surface of the stationary phase and therefore reduced the retention of tenuifolin. However, further increases in SDS concentration has shown no marked effect on the retention time of tenuifolin. 2.8% w/v SDS could provide a more appropriate elution time for separation and better retention factors for tenuifolin in Radix Polygalae. Therefore, 2.8% w/v SDS was used as the surfactant for further studies.

#### Selection of cosurfactant type and concentration

A cosurfactant, for instance, a short-chain alcohol was utilized to enhance and stabilize the microemulsion system. Moreover, cosurfactants play important role in phase behavior^24^. 1,2-propanediol, n-propanol and n-butanol were used as cosurfactants, with which the retention times for tenuifolin were found to be 24.01 min, 16.23 min and 9.66 min, respectively, and the symmetry factors were 0.57, 0.73 and 0.94, respectively. Therefore, the optimum cosurfactant was n-butanol in this study. Five concentrations of n-butanol were investigated (6.5, 7.0, 7.5, 8.0 and 8.5% v/v) (Fig. 3b). The retention time of tenuifolin was reduced upon increasing the concentration of n-butanol from 6.5 to 8.0% v/v, which was due to an increase in the solubilization capacity of the microemulsion with the use of n-butanol. Nevertheless, a further increase in the n-butanol concentration had no marked effect on the retention time. The use of 7.0% v/v n-butanol provided a better separation selectivity and suitable elution time for tenuifolin in Radix Polygalae. Therefore, 7.0% v/v n-butanol was chosen as the optimal cosurfactant concentration in subsequent experiments.

#### Selection of oil type and concentration

The type and concentration of the oil phase can significantly affect the retention time of analytes in the MELC method^25^. Three types of organic solvents (n-hexane, n-heptane and n-octane) were investigated in this study. However, n-hexane and n-heptane made forming stable microemulsions difficult. Therefore, n-octane was chosen as the oil type. Five concentrations of n-octane were investigated (0.4, 0.6, 0.8, 1.0 and 1.2% v/v) (Fig. 3c), which showed that the retention time of tenuifolin decreased as the n-octane concentration increased from 0.4 to 1.0% w/v. However, with a further increases in the n-octane concentration, the retention time of tenuifolin did not change significantly. The augmentation of n-octane can increase the content of organic reagents in the mobile phase and improve the elution ability. The n-octane molecule can also interfere with the surface of the stationary phase to reduce the polarity of the stationary phase and enhance the elution ability. A concentration of 0.8% v/v was suitable for routine use to separate tenuifolin in Radix Polygalae as it provides good peak efficiency and suitable elution time.

#### Selection of column temperature

**Figure 4:**
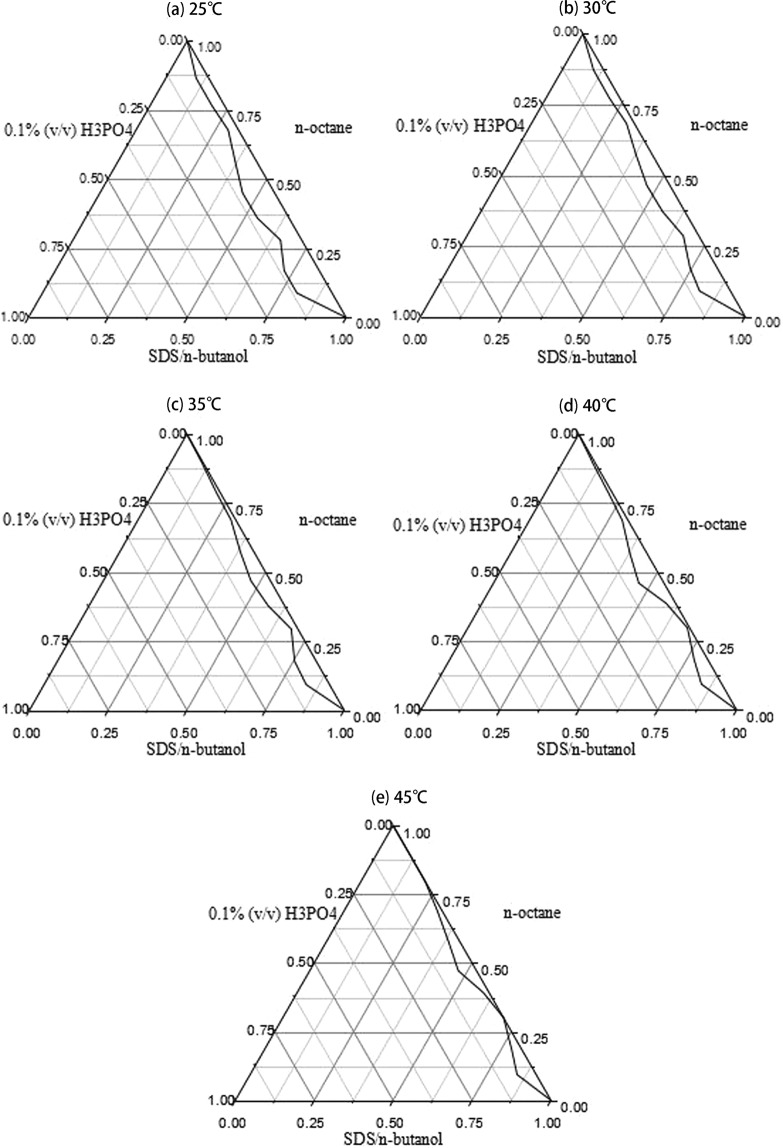
We select SDS and n-butanol as surfactant and cosurfactant, n-octane as oil phase, 0.1% H3PO4 as water phase to draw pseudo-ternary phase diagram. (**a**) A pseudo-ternary phase diagram of a W/O-type nanoemulsion region at 25 °C. (**b**) A pseudo-ternary phase diagram of a W/O-type nanoemulsion region at 30 °C. (**c**) A pseudo-ternary phase diagram of a W/O-type nanoemulsion region at 35 °C. (**d**) A pseudo-ternary phase diagram of a W/O-type nanoemulsion region at 40 °C. (**e**) A pseudo-ternary phase diagram of a W/O-type nanoemulsion region at 45 °C.

Five different column temperatures (25, 30, 35, 40 and 45 °C) were examined in this study (Fig. 3d). It was found that the retention time of tenuifolin was not significantly affected by temperature change. The result was in line with the findings reported^26^. However, the temperature has a significant effect on the area of the microemulsion region. With increases in the temperature, the area of the microemulsion region was reduced and the stability of microemulsion was decreased. Finally, to protect the chromatographic column and maintain the stability of microemulsion, 25 °C was selected from this study (Fig. 4).

### Validation of chromatographic parameters

#### Identification of tenuifolin

**Figure 5:**
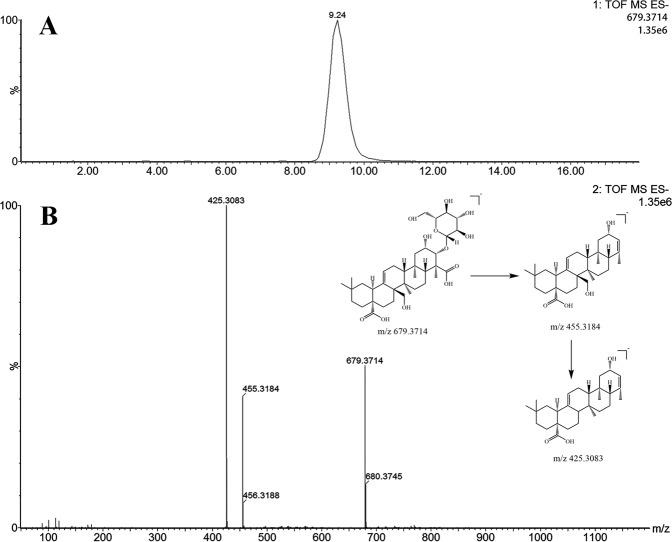
Extracted ion chromatograms (EICs) and MS/MS spectra of tenuifolin.

In order to identify the tenuifolin, an aliquot of 5 µL filtrate (48.00 µg/mL) was injected into the UPLC-Q-TOF-MSE system for analysis. The total ion current (TIC) chromatogram and MS/MS spectra including proposed fragmentation path ways of tenuifolin in negative modes are depicted in Fig. 5. Tenuifolin showed quasi-molecular ion [M-H] at m/z 679.3714 in negative ion mode, and yielded fragment ions at m/z 455.3184 and 425.3083 respectively. According to the accurate mass and its fragment information, it was identified as tenuifolin.

#### Selectivity, linearity and sensitivity

**Figure 6:**
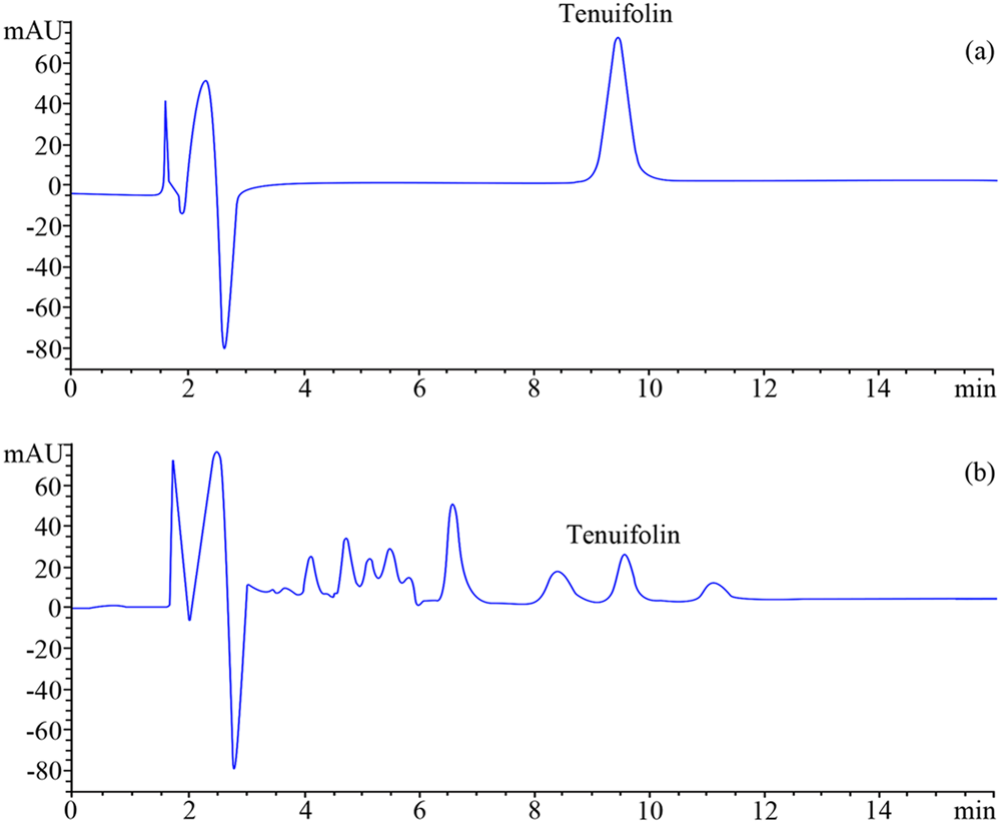
MELC chromatograms of (**a**) the standard solution and (**b**) the sample solution.

Selectivity was confirmed in the chromatograms of the standard solution and the sample solution. It is apparent that tenuifolin was clearly baseline separated in the sample solution, eluting in less than 10 min. The optimized separation is demonstrated in Fig. 6. Method linearity was evaluated in the range of 48.00–960.00 µg/mL. The regression equation was as follows Eq. (1):

1 $${\rm{y}}=169{\rm{.27x}}+31.32$$

where *y* is the peak area and *x* is the concentration (µg/mL) (Table 1). The linear regression analysis data showed a good linear relationship with a correlation coefficient (*r*) of 0.999. The LOD and LOQ values were 2.34 and 6.76 µg/mL, respectively, which showed high sensitivity under the experimental conditions.

**Table 1:** Linearity date of the MELC method.

Concentration (µg/mL)	Peak area
48 96	8799.28 16263.24
192	30356.71
400	69718.33
640	108229.02
960	162176.52

#### Precision and accuracy

The intraday and interday variations are summarized in Table 2. The RSD were less than 0.98% and 1.54% for intraday and interday variation, respectively. The average recoveries ranged from 96.23% to 99.28%. The RSD values were 0.41%, 1.39% and 1.36%, respectively (Table 3). The results suggested the feasibility of the MELC method for detecting tenuifolin in Radix Polygalae.

**Table 2:** Intra-day and Inter-day precision date of the MELC method.

Nominal concentration (µg/mL)	Intra-day		Inter-day	
	Amount found Mean (N = 5) ± SD	Precision (% RSD)	Amount found Mean (N = 15) ± SD	Precision (% RSD)
48.00	47.02 ± 0.47	0.98	47.55 ± 0.73	1.54
480.00	478.02 ± 1.93	0.40	477.80 ± 2.60	0.54
960.00	958.12 ± 2.16	0.23	958.43 ± 2.59	0.27

**Table 3:** Recovery of tenuifolin in Radix Polygalae.

Original amount (mg)	Spiked amount (mg)	Found amount (mg)	Recovery (%)	Average recovery (%)	RSD (%)
10.76	8.68	19.08	96.65		
10.68	8.68	18.92	95.88	96.23	0.41
10.64	8.68	18.91	96.15		
10.81	10.85	21.42	97.78		
10.89	10.85	21.41	96.97	98.13	1.39
10.72	10.85	21.53	99.63		
10.59	13.02	23.41	98.11		
10.66	13.02	23.76	100.75	99.28	1.36
10.68	13.02	23.59	98.97		

#### Repeatability and stability

For the repeatability study, the result showed that the value of RSD was 1.98%, which indicated that our method was reproducible. The stability was assessed by the same sample solution. The value of RSD for tenuifolin was 1.42%, indicating that the sample solution remained stable for at least 24 hours.

#### Comparison with conventional HPLC analysis

To prove the validity of the MELC method, the results obtained by use of the proposed method were compared with conventional HPLC analysis. A conventional HPLC gradient mobile phase of methanol-0.05% aqueous phosphoric acid (70:30) was employed. Compared with conventional HPLC analysis, the MELC method has a lower detection limit and higher sensitivity under identical experimental conditions^26^. The LOD of tenuifolin by way of HPLC was 3.50 µg/mL, and the LOD of tenuifolin with MELC was 2.34 µg/mL. The LOQ of tenuifolin with HPLC was 7.50 µg/mL, and the LOQ of tenuifolin in the MELC method was 6.76 µg/mL.

### Application of the method

The validated method was applied to determine the tenuifolin contents in ten different samples of Radix Polygalae collected from various regions of China. The MELC results showed that the content varied between 0.83 and 3.21%. The HPLC results showed that the content varied between 0.71 and 2.98%. The results of the two methods are consistent. Among them, the contents of tenuifolin in Radix Polygalae sample No. 2 were markedly higher than those in samples collected from other regions (Table 4).

**Table 4:** The content of tenuifolin in Radix Polygalae (N = 3).

No.	HPLC		MELC	
	Mean content (%)	RSD (%)	Mean content (%)	RSD (%)
1	2.41	0.98	2.82	0.87
2	2.98	1.32	3.21	1.12
3	2.03	1.14	2.62	1.04
4	1.86	1.09	2.14	0.79
5	2.29	0.88	2.27	1.07
6	0.71	1.17	0.83	1.12
7	0.87	1.24	0.92	1.76
8	1.39	1.67	1.47	0.81
9	1.41	1.91	1.29	1.22
10	1.21	1.31	1.50	0.91

## Conclusions

A sensitive, simple and efficient MELC method was developed for the rapid detection of tenuifolin in Radix Polygalae. Compared with HPLC, MELC method uses less organic solvent and offers a faster analysis time for the determination of tenuifolin with previous^10^. In addition, the proposed method is characterized by a high degree of sensitivity, precision, accuracy and separation. The method was successfully applied to samples of Radix Polygalae from different regions of China. In future research, the chromatographic conditions in this paper could be used to analyze tenuifolin in other biological samples, such as tissue homogenates, plasma and urine. Although the MELC assay is not a substitute for a more accurate and sensitive assay based on LC-MS/MS, the MELC method could provide a new choice for the routine quality control analysis of traditional Chinese medicinal materials as a powerful tool.

## Materials and Methods

### Chemicals

Sodium dodecyl sulfate (SDS), cetyltrimethyl ammonium bromide (CTAB) and Tween-80 were obtained from Sinopharm Chemical Reagent Co., Ltd. (Shanghai, China). N-butanol, n-propanol and 1,2-propanediol of HPLC grade were obtained from Oupo Bio-Technique Co., Ltd. (Shanghai, China). N-octane, n-hexane and n-heptane were all of HPLC grade and purchased from Bodi Chemicals Co., Ltd. (Tianjin, China). Tenuifolin (purity > 98.0%, lot number: 111849–201001) was purchased from Shijiazhuang QiDI Biological Technology Co., Ltd. (Shijiazhuang, China). Ten samples of Radix Polygalae were collected from Wanrong, Yuncheng, and Jiang counties of Shanxi Province and from Xinmi and Yuzhou Counties of Henan Province, and identified by Professor Xian-Hua Liu from the Anhui University of Chinese Medicine. All of the above reagents and chemicals were of analytical grade.

### Chromatographic conditions

The analyses were carried out using an Agilent Technologies 1100 HPLC system (USA) with a Welch Materials XB-C_18_ (4.6 × 250 mm, 5 μm) column. A microemulsion as the mobile phase was prepared by mixing 2.8% (w/v) SDS, 7.0% (v/v) n-butanol, 0.8% (v/v) n-octane and 0.1% (v/v) H_3_PO_4_. Before being injected into the HPLC system, the microemulsion mobile phase was filtered through 0.22 μm nylon membrane filters. During the analyses, the column temperature was set at 25 °C, and the injection volume was 10 μL. The flow rate of the mobile phase was controlled at 1.0 mL/min and peaks were detected at 210 nm^21,22^.

### Preparation of sample solutions

Approximately 1.0 g of Radix Polygalae powder (passing through a No. 3 sieve, 355 ± 13 µm) was extracted with 50 mL of 70% methanol in an ultrasonic bath for 1 h. After cooling, it was brought up to the initial volume with 70% methanol and then filtered through filter paper. The filtrate (25 mL) was transferred to a round bottom flask and evaporated to dryness. Then, 25 mL of 10% sodium hydroxide was added to the residue. The solution was heated and refluxed for 2 h, and the pH was adjusted to 4 with concentrated hydrochloric acid after cooling. The mixture was extracted by using 50 mL of n-butanol three times. The extraction solutions were collected and concentrated to dryness, dissolved with methanol and moved into a 25 mL volumetric flask^27^. Before being injected, the solution was filtered by 0.22 μm nylon membrane filters.

### Preparation of working standard solutions

A standard solution of tenuifolin was prepared by accurately weighing 60 mg of the analyte and dissolving it in a 50 mL volumetric flask using methanol. The standard solutions (1.20 mg/mL) were diluted directly to appropriate concentrations with methanol to produce the working standard solutions. All standard solutions were passed through 0.22 μm nylon membrane filters before analysis.

### Preparation of microemulsion

The microemulsion contained SDS (2.8%, w/v), n-butanol (7.0%, v/v) and n-octane (0.8%, v/v) as the surfactant, cosurfactant and oil phase, respectively. Briefly, a certain amount of SDS, n-butanol and n-octane were mixed in an ultrasonic bath for 10 min. Then, 0.01% (v/v) H_3_PO_4_ was added to the mixed system and mixed in an ultrasonic bath for 30 min.

### Particle size measurement of the mobile phase

The microemulsion mobile phase consisting of 2.8% (w/v) SDS, 7.0% (v/v) n-butanol, 0.8% (v/v) n-octane and 0.1% (v/v) H_3_PO_4_ was filtered through 0.22 μm filters. The reported size was the Z-average size (cumulant mean) of three replicates determined at 25 °C using a particle size analyzer (Malvern, UK).

### Method validation

Suitability of the optimized MELC method for the analysis of tenuifolin was evaluated with validation studies including specificity, linearity, sensitivity, limit of detection (LOD), limit of quantification (LOQ), repeatability, accuracy and stability of the sample solution.

#### Specificity, linearity and sensitivity

Specificity was evaluated by comparing the chromatograms of the standard solution and the sample solution. The linear dynamic range was selected within 48.00–960.00 µg/mL. Six different concentrations of tenuifolin were prepared at 48.00, 96.00, 192.00, 400.00, 640.00 and 960.00 µg/mL. The calibration curve was obtained over this range by plotting the peak area against the concentration of tenuifolin. Sensitivity was expressed by the LOD and LOQ, which were determined by signal-to-noise ratios greater than 3 and 10, respectively.

#### Precision and accuracy

Precision of the method was represented by the measurement of the intraday and interday variations of standard solutions at low (48.00 µg/mL), middle (480.00 µg/mL) and high (960.00 µg/mL) concentration levels. The variations were expressed as the relative standard deviations (RSD). Intraday variation was determined for five replicates on the same day. Interday variation was evaluated by analyzing five replicates on three successive days. The accuracy of the method was determined by recovery studies using the standard addition method. Known amounts of teuifolin at three levels (8.68, 10.85, and 13.02 mg) were spiked into approximately 0.50 g of Radix Polygalae powder sourced from Xinmi of Henan Province, and then extracted and analyzed as described above. Recovery was calculated by Eq. (2).

2 $$\begin{array}{cc}{\rm{recovery}}\,( \% ) & =\frac{{\rm{amount}}\,{\rm{found}}-{\rm{original}}\,{\rm{amount}}}{{\rm{amount}}\,{\rm{spiked}}}\end{array}\times 100 \% $$

#### Repeatability and stability

Repeatability was evaluated under optimum conditions by injecting six sample solutions at the same concentration that were extracted from the same batch of Xinmi. Stability was tested within the same sample solution stored at room temperature and assayed at 0, 2, 4, 8, 12 and 24 h.

## Data Availability

The datasets generated during and/or analyzed during the current study are available from the corresponding author on reasonable request.

## References

[1] Li J (2007). Simultaneous determination of phenols in Radix Polygalae by high performance liquid chromatography: quality assurance of herbs from different regions and seasons. J Sep Sci..

[2] Choi JG (2011). Polygalae Radix inhibits toxin-induced neuronal death in the Parkinson’s disease models. J Ethnopharmacol..

[3] Zhao H, Wang ZC, Wang KF, Chen XY (2015). Aβ peptide secretion is reduced by Radix Polygalae-induced autophagy via activation of the AMPK/mTOR pathway. Mol Med Rep..

[4] Shin JW (2017). Reduced consolidation, reinstatement, and renewal of conditioned fear memory by repetitive treatment of Radix Polygalae in mice. Front Psychiatry..

[5] Liu P (2010). Potential antidepressant properties of Radix Polygalae (Yuan Zhi). Phytomedicine..

[6] Chen LP, Wang FW, Zuo F, Jia JJ, Jiao WG (2011). Clinical Research on Comprehensive Treatment of Senile Vascular Dementia. Journal of Traditional Chinese Medicine..

[7] Qiu H (2016). Dihuang Yinzi, a classical Chinese herbal prescription, for Amyotrophic Lateral Sclerosis: a 12-year follow-up case report. Medicine (Baltimore)..

[8] Cao Q (2016). Tenuifolin, a saponin derived from Radix Polygalae, exhibits sleep-enhancing effects in mice. Phytomedicine..

[9] Liu YM (2015). Tenuifolin, a secondary saponin from hydrolysates of polygala saponins, counteracts the neurotoxicity induced by Aβ_25-35_ peptides *in vitro* and *in vivo*. Pharmacol Biochem Behav..

[10] Li J (2007). HPLC determination of total saponins in Radix Polygalae. Chin. J. Pharm Anol..

[11] Hao ZY, Wang J, Zhang YM, Chen AJ (2011). Determination of tenuifolin in Polygalae Radix from different regions by RF-HPCE. J. Chin Pharm Sci..

[12] Ma B (2014). Quantitative analysis of tenuifolin concentrations in rat plasma and tissue using LCMS/MS: application to pharmacokinetic and tissue distribution study. J Pharm Biomed Anal..

[13] Anđelija M, Darko I, Biljana SJ, Mirjana M (2009). Robustness Testing of Microemulsion Liquid Chromatographic Separation of Simvastatin and its Impurities. Journal of Liquid Chromatography & Related Technologies..

[14] Li L, Lai C, Xuan X, Gao C, Li N (2016). Simultaneous determination of hydrochlorothiazide and losartan potassium in osmotic pump tablets by microemulsion liquid chromatography. J Chromatogr Sci..

[15] Malenović A, Jančić-Stojanović B, Ivanović D, Medenica M (2010). Forced degradation studies of simvastatin using microemulsion liquid chromatography. Journal of Liquid Chromatography & Related Technologies..

[16] Altria KD, Marsh A, Clark BJ (2006). High performance liquid chromatographic analysis of pharmaceuticals using oil-in-water microemulsion eluent and monolithic column. Chromatographia..

[17] Hammouda ME, Abu El-Enin MA, El-Sherbiny DT, El-Wasseef DR, El-Ashry SM (2015). Simultaneous determination of enalapril and hydrochlorothiazide in pharmaceutical preparations using microemulsion liquid chromatography. J Chromatogr Sci..

[18] Momenbeik F, Roosta M, Nikoukar AA (2010). Simultaneous microemulsion liquid chromatographic analysis of fat-soluble vitamins in pharmaceutical formulations: optimization using genetic algorithm. J Chromatogr A..

[19] Abou-Taleb NH, El-Wasseef DR, El-Sherbiny DT, El-Ashry SM (2015). Multiobjective optimization strategy based on desirability functions used for the microemulsion liquid chromatographic separation and quantification of norfloxacin and tinidazole in plasma and formulations. J Sep Sci..

[20] Gao H, Huang H, Zheng A, Yu N, Li N (2017). Determination of quantitative retention-activity relationships between pharmacokinetic parameters and biological effectiveness fingerprints of Salvia miltiorrhiza constituents using biopartitioning and microemulsion high-performance liquid chromatography. J Chromatogr B Analyt Technol Biomed Life Sci..

[21] Huang H (2014). Optimization of liquid chromatographic method for the separation of nine hdrophilic and hydrophobic components in Salviae miltiorrhizae Radix et Rhizoma (Danshen) using microemulsion as eluent. J Chromatogr B Analyt Technol Biomed Life Sci..

[22] Song R, Zhou J (2015). Microemulsion liquid chromatographic method for simultaneous separation and determination of six flavonoids of Apocynum venetum leaf extract. J Chromatogr B Analyt Technol Biomed Life Sci..

[23] Marsh, A., Clark, B. & Altria, K. Oil-in-Water Microemulsion High Performance Liquid Chromatographic Analysis of Pharmaceuticals. *Chromatographia*. **59** (2004).

[24] Garti N, Yaghmur A, Leser ME, Clement V, Watzke HJ (2001). Improved Oil Solubilization in Oil/Water Food Grade Microemulsions in the Presence of Polyols and Ethanol. J. Agric Food Chem..

[25] Mason TG, Wilking JN, Meleson K, Chang CB, Graves SM (2006). Nanoemulsions: formation, structure, and physical properties. Journal of Physics: Condensed Matter..

[26] Althanyan MS, Assi KH, Clark BJ, Hanaee J (2011). Microemulsion high performance liquid chromatography (MELC) method for the determination of terbutaline in pharmaceutical preparation. J Pharm Biomed Anal..

[27] National pharmacopoeia commission. *Chinese Pharmacopoeia*. 156–157 (2015).

